# Spontaneous Reporting of Adverse Drug Reactions in a Pediatric Population in a Tertiary Hospital

**DOI:** 10.3390/jcm10235531

**Published:** 2021-11-26

**Authors:** Laura López-Valverde, Èlia Domènech, Marc Roguera, Ignasi Gich, Magí Farré, Carlos Rodrigo, Eva Montané

**Affiliations:** 1 Department of Pharmacology, Therapeutics and Toxicology, Universitat Autònoma de Barcelona, 08193 Cerdanyola del Vallès, Spain; laura261lv@gmail.com (L.L.-V.); igichs@santpau.cat (I.G.); mfarre.germanstrias@gencat.cat (M.F.); 2 Department of Pediatrics, Hospital Universitari Germans Trias i Pujol, 08916 Badalona, Spain; eliadomenech.germanstrias@gencat.cat (È.D.); mroguera.germanstrias@gencat.cat (M.R.); crodrigo.germanstrias@gencat.cat (C.R.); 3 Department of Clinical Pharmacology, Hospital Universitari Sant Pau, 08025 Barcelona, Spain; 4 Department of Clinical Pharmacology, Hospital Universitari Germans Trias i Pujol, 08916 Badalona, Spain; 5 Department of Pediatrics, Obstetrics and Gynecology, Preventive Medicine and Public Health, Universitat Autònoma de Barcelona, 08193 Cerdanyola del Vallès, Spain

**Keywords:** adverse drug reaction, spontaneous reporting, pharmacovigilance, children, pediatrics

## Abstract

The pediatric population is a vulnerable group for adverse drug reactions (ADRs), and data on spontaneous reporting of ADRs in the hospital setting are scarce. We conducted a retrospective analysis of ADRs in pediatric patients spontaneously reported by health care professionals to a Pharmacovigilance Program in a tertiary hospital between 2010 and 2020, and we compared characteristics of ADRs between pediatric age subgroups. From 1787 spontaneously reported ADRs in an 11-year period, 103 (5.85%) were pediatric ADRs. The median age of patients with ADRs was 8.4 years (range 1 day–17 years) and 57.3% were male. The most frequent ADRs reported were nervous system disorders (13.6%) and the most frequently involved drugs were antineoplastics and immunodulators (32.4%). A 59.2% of the ADRs were serious and 55.3% were classified as being type B reactions. Medication errors were involved in 7.8% of the ADRs and 11.9% of the suspected drugs were used off-label. Spontaneous reports of ADRs in newborns, infants, and toddlers were more serious and less often described in the product data sheet than in children and adolescents (*p* < 0.001 and *p* = 0.004 respectively). Medication errors were more frequent in patients under two years of age. These results should be interpreted with caution due to under-reporting and biases in spontaneous reporting of ADRs.

## 1. Introduction

The definition of adverse drug reactions (ADRs) has been modified over time. In the early 1970s, the World Health Organisation (WHO) defined them as “any response to a drug that is noxious and unintended and that occurs at doses normally used in humans for the prophylaxis, diagnosis or treatment of disease, or for the modification of physiological function” [1]. Subsequently, the last European pharmacovigilance legislation (Directive of the European Parliament and of the Council 2010/84/EU), widened the definition of ADR to “A response to a medicinal product which is noxious and unintended”, which includes drug misuse, drug abuse, medication errors, and off-label use [2].

ADRs are a significant health problem both for individuals and for society as they are considered an important cause of morbidity and mortality, have a significant healthcare cost and have a negative impact on the quality of life of those who suffer from them [3]. ADRs account for 5% of hospital admissions, and occur in 10% of outpatients and in 10–20% of inpatients [4,5]. Furthermore, they are recognized as the fifth leading cause of in-hospital death [6].

The pediatric population is a vulnerable group to ADRs for several different reasons. Information from clinical trials about the safety and/or efficacy of medicines for pediatric use is often limited or even absent. Consequently, several drugs are prescribed off-label, leading to an increased risk of ADRs [7]. It is also common to consider this population as small adults, without taking into account the physiological changes they undergo from birth to adolescence and the immaturity of the organ systems involved in drug absorption, distribution, metabolism and excretion. This misjudgment leads to many medication errors in this age group, such as dosing and administration errors [8].

Over the years, several observational studies assessing pediatric ADRs have been conducted and included in numerous systematic reviews and meta-analyses. Overall incidences of ADRs were found to be around 10% in hospitalized children, 2% in hospital admissions and 1–1.5% in outpatient children [9,10]. Likewise, 12.3% of ADRs in hospitalized children and 39.3% of those in child hospital admissions were severe [9]. With regards to the characteristics of ADRs, the skin and gastrointestinal system were the most affected organs, and anti-infectives, antiepileptics, and non-steroidal anti-inflammatory drugs (NSAIDs) were the most frequently involved drugs [10,11]. Female sex, polymedication, comorbidity, and off-label use of drugs were identified as the main risk factors associated with ADRs in this population [9,10,12].

The spontaneous reporting system of suspected ADRs is the method most widely used in pharmacovigilance to generate signals or alerts and to recognize new safety concerns. Its main advantages include its simplicity and low cost; however, its most recognized disadvantages are under-reporting and its inability to calculate rates [13,14,15]. Under-reporting leads to a decrease in the sensitivity of the method, with signal detection delays being very common [14]. Priority ADR notifications are those involving newly marketed drugs and those undergoing additional follow-up, ADRs that have not been previously reported, serious ADRs, and those affecting pediatric population [15]. 

The characteristics of pediatric ADR reported in national or regional systems of pharmacovigilance such as the European or Spanish Pharmacovigilance Systems have already been published [16,17]. However, ADR data from pharmacovigilance systems in hospitals are scarce, and even absent for the pediatric population, despite the fact that the reporting of ADRs in these institutions provides valuable information on the use of their medicines.

The aims of the present study were to determine the characteristics of ADRs in pediatric patients registered in the spontaneous-reporting Hospital Pharmacovigilance Program database over an 11-year period and to compare them between different pediatric subgroup ages.

## 2. Materials and Methods

We followed the STROBE Statement when reporting the following study sections and their content [18].

### 2.1. Study Setting and Design

The Germans Trias i Pujol Hospital is a tertiary care hospital with 511 beds for a population of about 850,000 people living in the Barcelonès Nord I Maresme area of Barcelona, in Catalonia, Spain. During the study period, the hospital had an inpatient area with 40 pediatric beds and a neonatal intensive care unit with 5 beds; annually, there were 2000 admissions, 28,000 emergency department visits, and 25,000 outpatient visits.

The study was approved by the Research Ethics Committee of the Germans Trias i Pujol Hospital in February 2016. The study was conducted in 2021.

The Hospital Pharmacovigilance Program includes the spontaneous reporting method, in which ADRs are reported either by nurses, doctors, medical students, pharmacists, or clinical pharmacologists at the hospital. The ADRs come from any patient visiting the hospital, such as patients attending the emergency department, hospitalized patients, and outpatients followed up by hospital specialists. A spontaneous report of an ADR includes the following information: patient identification data, name of the suspected drug, description of the adverse reaction, and identification data of the reporter. Subsequently, for each reported case, clinical pharmacologists collect all the detailed data included in the yellow card in order to evaluate their causality attribution. All the reported ADRs are accurately evaluated by the Pharmacovigilance Committee, composed of clinical pharmacologists and specialized nurses. When cases are considered possible, probable, or definite, the ADRs are included in the registry.

We conducted a retrospective analysis of all ADRs spontaneously reported by health professionals in the Germans Trias I Pujol hospital. The ADRs were registered in the database of the Pharmacovigilance Program over 11 years, between 1 January 2010 and 31 December 2020.

### 2.2. Participants

All ADRs recorded in the Pharmacovigilance Program database were selected, and all patients aged ≤ 17 years were included in the study. All the included cases were previously reported to the Spanish System of Pharmacovigilance. 

### 2.3. Variables

The following information was extracted for each ADR case from the registry: year reported, age and sex of the patient, suspected drugs, number of involved drugs, indications, doses, route of administration and duration of the involved drug, drugs ‘under additional monitoring’, inappropriate drugs, drug–drug interactions, in-patient or outpatient setting, the system organ class related to the ADR, date of onset and end of the reaction, unlabeled reactions, mechanism of the ADR, severity of the ADR, outcome, medication errors, and ADRs of pharmacovigilance interest.

The following additional variables were available for patients included in the database from January 2016: duration of treatment, management of the involved drug, off-label prescription drugs, number of concomitant drugs and polymedication.

#### Variable Definitions and Classifications 

ADRs: Descriptive terms of reactions were classified by System Organ Classes (SOC) according to the MedDRA dictionary (Medical Dictionary for Regulatory Activities) [19].

ADRs of pharmacovigilance interest: Reported reactions meeting any of the priority criteria for reporting reactions were considered of interest for pharmacovigilance [20]. These criteria were: newly marketed drugs or drugs undergoing additional monitoring, reactions not previously reported, and serious ADRs (including causing the death of the patient, being life-threatening, leading to hospitalization or stay prolongation, causing a significant or persistent disability, or causing a congenital anomaly or birth defect).

Age subgroups of the pediatric population: Four groups were defined according to the International Conference on Harmonisation (ICH) guideline E 11: newborn (≤27 days), infants and toddlers (28 days to 23 months), children (2 to 11 years) and adolescents (12 to 17 years) [21]. Numerical age was established in years and with whole numbers from 0 to 17. Patients with an age between birth and six months were counted as 0 years. Similarly, patients aged between 7 and 12 months were counted as 1 year.

Drug classes: Suspected drugs were classified according to the categories of the Anatomical Therapeutic Chemical (ATC) classification System (level 1) [22].

Drug–drug interactions: If a drug–drug interaction was suspected, a review of the literature was done to document the interaction. Drug–drug interactions were classified as either pharmacodynamic or pharmacokinetic. Pharmacodynamic interactions were defined as those in which drugs influence each other’s pharmacologic effects, and were evaluated as either synergistic or antagonistic. Pharmacokinetic interactions were defined as those in which a drug may result in the increase or decrease of plasma drug concentrations [23].

Drug safety alerts: Alerts issued from the Spanish Medicines Agency were searched for on its website [24] and were correlated to the ADR registered in the database. These alerts were classified according to whether they were generated before or after the spontaneous reporting. When the notification preceded the drug safety alert, the time between the notification and the alert was recorded.

Drugs ‘under additional monitoring’: Drugs were classified as being or not being ‘under additional monitoring’. ‘Additional monitoring’ is a term denoted by the European Medicines Agency to medicines that are more intensively monitored than others [25]. This is generally because there is less safety information available—for example because the medicine has been recently marketed or there is limited data on its long-term use. A drug with additional follow-up is a drug that had an inverted black triangle (▼) on the package leaflet. A drug was denoted as ‘under additional monitoring’ if it was included in the EMA’s list of medicines under additional monitoring, according to the year in which the ADR occurred [26]. 

Duration of treatment: Refers to how long a patient was treated before the ADR onset. This was classified as ‘acute’ when the involved drug was started within the week before the beginning of the ADR, ‘subacute’ when it was started between 1 week and 6 months before the ADR and ‘chronic’ when the suspected drug was started more than 6 months before the ADR. In cases where two or more drugs with different starting times were involved, the shortest duration category was selected.

Inappropriate drugs for children: Drugs were classified as ‘inappropriate’ according to the KIDs List, defined as drugs or classes of drugs that should generally be avoided in people under 18 years of age because they pose an unnecessarily high risk to children and a safer alternative exists [27]. Ineffectiveness of drugs was not a criterion for this list. Two recommendations are used in the KIDs List: avoid and caution.

Mechanism of ADRs: ADRs were classified as type A or type B. Type A ADRs (augmented) result from an increase in the pharmacological action of the drug and depend on the mechanism of action of the drug; they are usually predictable, frequent, dose-dependent and have low mortality. Type B ADRs (bizarre) are not related to the mechanism of action of the drug, such as idiosyncratic or allergic hypersensitivity reactions; they are usually unpredictable, infrequent and have high mortality [28].

Medication error: A medication error was defined according to the National Coordinating Council for Medication Error Reporting and Prevention: ‘medication error is any preventable event that may cause or lead to inappropriate medication use or patient harm while the medication is in the control of the health care professional, patient, or consumer. Such events may be related to professional practice, health care products, procedures, and systems, including prescribing, order communication, product labeling, packaging, and nomenclature, compounding, dispensing, distribution, administration, education, monitoring, and use’ [29].

Off-label drug use: Off-label prescribing occurs when a drug is prescribed for an indication, a route of administration, or a patient group that is not included in the approved product information document for that drug [30]. Off-label use was classified as by “indication”, by “posology”, by “route of administration”, or by “patient age”.

Polymedication: Defined as when patients received more than five drugs [31].

Seriousness of ADRs: A serious ADR was defined according to the International Conference on Harmonisation (ICH) guideline E 2 D, which encompasses ADRs that are fatal, life-threatening, requiring hospital admission or prolongation of hospital stay, causing persistent or significant disability/incapacity, congenital anomaly/congenital defect or being otherwise medically important. The remaining cases were defined as non-serious ADRs [32].

Unlabeled reaction: Defined as when information on the observed reaction was not found in the product data sheet of the suspected medicine, which was obtained from the website of the Spanish Agency of Medicines and Medical Devices (AEMPS) [33].

### 2.4. ADR Causality Assessment

The Drug Safety Committee of the Hospital was responsible for assessing the causality attribution of all spontaneously reported ADRs in the Hospital Pharmacovigilance Program. Each reported case was evaluated in detail by clinical pharmacologists using the modified Karch and Lasagna algorithm that is used by the Spanish Pharmacovigilance System [34]. This algorithm assesses the following five items: temporal relationship between onset of drug administration and onset of the reaction, knowledge of the reaction in the literature, the clinical effects of withdrawal of and re-exposure to the drug involved, and assessment of alternative causes and background clinical factors that may have contributed to the onset of the reaction [34]. ADRs were included in the database if the Drug Safety Committee scored them as ‘possible’, ‘probable’ or ‘definite’. 

### 2.5. Statistical Analysis

For descriptive analysis we used the number of cases and percentages for categorical variables; median and range for ordinal variables; and mean and standard deviation (SD) for continuous variables. 

To compare the characteristics of ADRs between age subgroups, we decided to pool the age subgroups that included the newborns, infants and toddlers (NIT subgroup) due to the small sample size of each one, in order to assess statistical differences between pediatric subgroups. Children under two years of age are usually treated with similar drugs because they present with similar diseases that lead to hospital admission, and the nursing staffs that care for them are also similar.

The Pearson chi-square test was used for categorical variables and the Kruskall–Wallis test for ordinal variables. A bilateral *p*-value < 0.05 was used to determine statistical significance. 

Statistical analysis was performed using the SPSS statistical software package for Windows, version 15.0 (SPSS Inc., Chicago, IL, USA). 

## 3. Results

During the 11-year study period, a total of 1787 spontaneous ADR cases were recorded in the Pharmacovigilance Program database. A total of 103 (5.8%) suspected ADRs occurred in 92 pediatric patients and were subsequently included in the study (eight patients had two ADRs each, and one patient presented with four ADRs).

A total of 90.3% of ADRs (93 cases) were reported by medical doctors (including clinical pharmacologists), 4.8% of ADRs (5 cases) by nurses, 3.9% of ADRs (4 cases) by medical students and 0.97% (1 case) by pharmacists. The number of ADRs reported annually ranged from two to fifteen, with an average of nine notifications per year. Between 2010 and 2014, 37 suspected ADRs (36%) were reported, and between 2016 and 2020, 58 cases (56.4%) involving 84 drugs were reported. 

The mean age of patients was 8.4 years (SD 6.2), ranging 0–17 years (median 10 years), of which 57.3% (59 cases) were males. 

### 3.1. Characteristics of ADRs 

The most frequent ADRs were nervous system disorders (14 cases; 13.6%), disorders of the immune system and disorders of the skin and subcutaneous tissue (12 cases; 11.7% each). Skin rash or erythema (9 cases), infusion reactions (7 cases) and arrhythmias (6 cases) were the most often described ADRs. The remaining types of ADR are detailed in Table 1.

A total of 59.2% (61 cases) of ADRs were serious (Table 1). A total of 44.7% (46 cases) were type A ADRs and 55.3% (57 cases) were type B ADRs. A total of 40.8% (42 cases) of ADRs were hospital-acquired, 34% (35 cases) led to hospital admission and the remaining occurred in outpatient clinics.

A total of 67 pediatric ADRs (65%) were considered to be of pharmacovigilance interest.

### 3.2. Characteristics of Suspected Drugs

The median number of suspected ADR drugs was 1.4 (range 1–6). In 24.2% (25 cases) of ADRs there was more than one involved drug (two drugs in twenty-one ADRs, four in two ADRs, five in one ADR and six drugs in another).

In total, there were 139 suspected drugs for 103 ADRs. In 13 (12.6%) ADRs, a drug–drug interaction was present; all of the interactions were pharmacodynamic and synergistic interactions. Of these, 54% (7 cases) were combinations of immunosuppressants and/or antineoplastics. 

Forty-five ADRs involved drugs (32.4%) that are classified as ATC category L (Antineoplastic agents and immunomodulators), 30 (21.6%) as category N (Nervous system), 22 (15.8%) in the category J (Anti-infectives for systemic use), and 12 (8.6%) as category R (Respiratory system; Table 2).

The most frequently involved drugs were ibuprofen (in eight cases; 5.8%), methotrexate (in seven cases; 5%) and adalimumab, azathioprine, and salbutamol (in five cases each; 3.6% each; Table 2).

Medication errors were observed in 7.8% (8 cases) of ADRs, half (4 patients) were younger than 6 months. Accidental overdose occurred in 7 cases, and in one patient, immunoglobulin was administered instead of serum albumin (Table 3). All patients recovered. Of all involved drugs, in 7.9% (11 cases) of cases the reaction was unlabeled. Nine drugs (6.5%) were under ‘additional monitoring’; all of these drugs were recently marketed except for two cases involving valproic acid. Four drugs (3.9%) were considered potentially inappropriate for the pediatric population according to the KIDs list, which recommends avoiding the drug in three cases (treated with valproic acid, lidocaine and haloperidol) and drug caution in one case (treated with acetylsalicylic acid).

Three suspected ADRs (2.2%) were reported to the Spanish Pharmacovigilance System before safety alerts were issued with the same drug–reaction pair. The first notification was that of a patient with liver failure due to acetaminophen overdose reported in January 2012, and two months later an alert was issued on dosing errors in children associated with intravenous acetaminophen [35]. In 2015, a patient with a vitamin D overdose was reported, and four years later an alert was issued on the risk of vitamin D overdose in children resulting in hypercalcemia [36]. The third notification, reported in 2017, was that of a patient with a psychiatric disorder two weeks after starting isotretinoin, and a year later, an alert was issued on the risk of neuropsychiatric disorders associated with isotretinoin and other retinoids [37].

The median number of concomitant drugs prescribed per ADR was four (range 1–19). In 77.6% of ADRs (45 cases) there were at least five concomitant drugs, in 13.8% (8 cases) between 6 and 10 drugs, and in 8.6% (5 cases) at least 11 concomitant drugs. In 13 cases (22.4%), patients were considered polymedicated. The involved drug was started within the week before the ADR in 33 patients (41.8%), of whom 11 patients received anti-infectives for systemic use; the duration of the involved treatment was ‘subacute’ in 42 patients (53.2%) and ‘chronic’ in 4 (45.1%), of whom 31 and 4 patients received antineoplastic and immunomodulatory agents, respectively. 

Off-label use was observed in 17.2% of the ADRs (10 cases) and in 11.9% of the involved medications; 5 cases were off-label by patient age, 3 cases by indication, 1 case by patient age and dose, and 1 by patient age and indication (Table 4).

The involved drug was withdrawn in 33 cases (41.8%), its dose was reduced in 5 cases (6.3%) and in 41 cases (51.9%) no modification of the therapeutic regimen was carried out.

A total of 89.3% (92 cases) of patients completely recovered from their ADRs. Two patients had sequelae (1.9%), one with skin sequelae due to cellulitis after DTaP vaccine administration and the other with neurological sequalae due to methotrexate-induced leukoencephalitis. Three patients died (2.9%) due to pre-existent comorbidities, with the drug having a contributory role; consequently, these deaths were not avoidable. In the first case, ibuprofen-induced pulmonary hemorrhage in a very preterm newborn; in the second case, bupivacaine and epinephrine-induced ventricular arrhythmia in an infant aged 1 month, whose autopsy revealed that the patient had a congenital heart disease consisting of a deposition cardiomyopathy; and in the third patient, ondasetron induced a cardiac arrest in a five year old boy, whose molecular autopsy revealed that the patient had genetic mutations probably associated to early repolarisation syndrome and idiopathic ventricular fibrillation.

### 3.3. Comparison of ADRs between Age Groups 

The neonates, infants and toddlers subgroup had a significantly higher proportion of serious ADRs (*p* < 0.001) and of unlabeled reactions (*p* = 0.004) than the subgroups belonging to children and adolescents. The most frequent type of ADR and involved drugs were different between the age subgroups, with nervous system disorders and nervous system drugs more frequent in neonates, infants, and toddlers; immune system disorders and antineoplastic and immunomodulatory drugs more frequent in children; and skin disorders and antineoplastic and immunomodulatory drugs more frequent in adolescents (Table 5). 

## 4. Discussion

Of the total number of spontaneously reported ADRs in the hospital pharmacovigilance database, 5.8% occurred in the pediatric population; this proportion was in line with the rate of pediatric beds in the hospital (which represents about 7.5% of their total beds), and was lower than that reported in the European pharmacovigilance database (EudraVigilance) [16] (11.2%), but similar to that reported in the Spanish pharmacovigilance database (FEDRA) [17] and the worldwide pharmacovigilance database (VigiBase) [38]. 

Reports of ADRs by gender were slightly higher in males, similar to VigiBase, but contrary to the FEDRA and EudraVigilance databases, where ADRs are predominantly reported in females. The number of suspected ADRs reported in the last years of the study period increased compared to the first years (2010–2014: 36% vs 2016–2020: 56%), due to the incorporation of pediatricians into the hospital pharmacovigilance program from 2013, and their active involvement in the reporting of ADRs. 

The proportion of serious ADRs was higher than that previously reported by FEDRA [17]. This difference may be due to the fact that FEDRA also receives reports from primary healthcare settings, in which ADRs are generally milder than those reported in hospitals. The same reason could also explain why type B ADRs were more frequent than type A ADRs. In addition, a bias in the spontaneous reporting database is to be expected, as type B adverse reactions are of greater interest due to their unpredictable and infrequent nature [39]. 

Among the most frequently reported ADRs were immune system disorders, and among the involved drugs, the most frequently reported were antineoplastic and immunomodulatory agents. This could be due to the close follow-up of pediatric patients treated with selective immunosuppressants for autoimmune diseases in our center. In contrast, national or regional spontaneous reporting databases were dominated by vaccines and vaccine-related effects [16,17,38] and reviews of observational studies by gastrointestinal disorders and anti-infectives for systemic use [10,11]. 

There are many useful algorithms for assessing causality in ADRs, often used by medical journals and national pharmacovigilance organizations. Although the most frequently used causality systems worldwide are the Naranjo algorithm and the WHO-UMC criteria [40,41], we used the modified Karch and Lasagna algorithm because the members of the Drug Safety Committee of the Hospital have experience and training in this algorithm, which is used by the Spanish Pharmacovigilance System [34]. Two thirds of ADRs were considered to be of pharmacovigilance interest, which is an indicator of the high-quality reporting of the ADRs collected in the database. In line with the above, the Spanish Medicines Agency generated three safety alerts from three cases coming from our database; therefore, these facts suggest that the Hospital Pharmacovigilance Program contributed to the generation of these safety alerts, highlighting the importance of reporting and recording ADRs occurring in the hospital setting.

Medication errors were observed in almost 8% of pediatric ADR reports—most were serious and occurred in newborns and infants. It is well known that, in general, medication errors are highly underreported. Many barriers to reporting have been described, including fear of disciplinary actions, fear of a lack of confidentiality, lack of time, and lack of awareness that an error has occurred. Although our reporting system is non-punitive, the reporting rate of medication errors was low, with most of them being serious [42]. Most observational studies exclude medication errors by using the WHO definition of ADRs, contrary to studies concerning spontaneous reporting databases, that have warned of the importance of medication errors among very young children. In the VigiBase database, errors also predominated in newborns and infants, and were associated with accidental overdose [38]. In the EudraVigilance database, pediatric ADR errors were mainly associated with inappropriate dose and indication [16]. 

Off-label use was observed in more than 10% of the involved drugs, and in 17% of ADRs—more than half of which were serious. This low rate may partly be explained by the fact that pediatricians in our hospital are very aware and avoid prescribing off-label drugs as much as possible, especially when evidence of efficacy in children is lacking. Off-label use of drugs is a known risk factor for pediatric ADRs [43,44]. In fact, the risk of ADRs has been reported to be more than three times higher in children who receive off-label drugs in the hospital setting [10]. Other studies have reported that 30% of the drugs causing pediatric ADRs are off-label drugs with respect to dose, indication or patient age [9]. Spontaneous reporting databases have also reported relevant off-label use of drugs, such as EudraVigilance, where the majority of non-vaccine-related pediatric ADRs were associated with off-label use in the pediatric population [16]. 

Medication errors and off-label use of drugs in the pediatric population are concerns in the hospital setting. To improve pediatric patient safety, programs could be created to inform, educate and motivate families in the prevention of these recurrent problems in the pediatric population; the continuous training of healthcare professionals should also be reinforced.

Drugs considered potentially inappropriate for prescribing in all or in a select subgroup of pediatric patients were involved in almost 4% of the ADRs, according to the KIDs list. This is a recently published evidence-based list of medicines that are recommended to be avoided or, if this is not clinically possible, to be used with caution and accompanied by appropriate monitoring [27]. Due to its recent publication, no other studies have been identified that have used this list with which to compare our results. In addition, some drugs are not on this list because ineffectiveness is not a criterion for inclusion or because there is insufficient evidence to confer a clear safety risk when using therapeutic doses in children, such as over-the-counter pediatric cough and cold preparations; however, we consider some of these to be potentially inappropriate.

One of the most relevant results of this study was the differences found between the pediatric age subgroups. The subgroup with the most serious ADRs was the newborns, infants, and toddlers group, similar to what is observed in the FEDRA database [17]. This could be explained by the fact that this youngest pediatric subpopulation has immature organs, mainly the kidney and liver, which affects the pharmacokinetic profile of drugs; therefore, the processes of drug absorption, distribution, metabolism and excretion occur more slowly, leading to higher tissue drug concentrations [45]. However, in children and adolescents, these pharmacokinetic processes are more accelerated than in the adult population. In addition, newborns, infants and toddlers was the subgroup with the most unlabeled ADRs, probably due to a lack of safety and efficacy information in this subgroup of the population. To mitigate the complexity of drug management in younger pediatric patients, joint action between pharmacovigilance programs with clinical pharmacologists and pediatric and obstetric clinical services is needed.

As in the FEDRA database, the comparison between the pediatric subgroups showed differences in the most frequent disorders and drugs involved; however, these results differed. In our study, nervous system and blood disorders and nervous system drugs predominated in neonates, infants and toddlers; while immune system and skin disorders, and antineoplastic and immunomodulatory agents were more frequent in children and adolescents. In contrast, in the FEDRA database, congenital disorders and alimentary tract drugs predominated in newborns, general disorders and respiratory drugs were prominent in infants and children, and nervous system disorders and drugs were prominent in adolescents [17]. This could be due to differences between drugs used in the hospital setting and in the primary care system.

The ultimate goal of a hospital pharmacovigilance program is to improve medication safety and prevent harm. Despite the limitations of the spontaneous ADR reporting system, it is useful for monitoring internal trends and providing signals on drug safety that could be addressed through internal policies [46]. Some actions that could help improve drug safety in hospitals are aimed at promoting consultation with clinical pharmacologists for the evaluation and diagnosis of the causality of ADRs, monitoring of drug doses, safe prescription tools such as electronic prescriptions to assist in avoiding errors and drug interactions to prevent ADRs, double-checking the administration of risky medications, and having nurses with experience in working with hospitalized pediatric patients.

### Strengths and Limitations

This study has several limitations. The main limitation relates to the under-reporting of ADRs inherent in spontaneous reporting. This means that cases reported spontaneously to the pharmacovigilance program generally represent only a small of the number of those that have actually occurred [14]. Several factors have been associated with this under-reporting problem, such as lack of motivation and time of reporters, or the absence of an effective pharmacovigilance program with protocols and tools that are easy to use [13,47,48]. However, the effect of underreporting can be somewhat lessened if submitted reports, irrespective of number, are of high quality [49]. The information in spontaneous reports of ADRs is subject to the possible influence of a number of biases, such as the detail and quality of the reported data, and the length of time a product has been on the market [49]. This potential data quality bias could be addressed because the data were collected by clinical pharmacologists, thus ensuring high quality data. As reports of all drugs were included, the bias resulting in maximal reporting of ADRs in the second year of marketing of a drug could also be controlled for. Thus, only 6.5% of suspected drugs were under ‘additional monitoring’. Furthermore, in the absence of data on the number of patients exposed to a medicine, estimation of the exact incidence of pediatric ADRs cannot be obtained through spontaneous reporting [13,14]. It should be noted that since this study was conducted in a hospital setting, reported cases of ADRs may differ from unreported cases in terms of severity [50]. The fact that we present a specific hospital pharmacovigilance program with own characteristics may lead to a bias that makes it difficult to extrapolate the results and compare them robustly with pharmacovigilance programs of other hospitals. 

This study also has strengths. This is the first study identified in Europe based on a hospital spontaneous reporting program of ADRs in a pediatric population, with very detailed information from cases that allowed us to collect several variables not included in previous national database reporting studies. It is important to note that the quality of ADRs was quantified according to their relevance for pharmacovigilance and the suitability of drugs for the pediatric population was assessed according to the KIDs list—a list of potentially inappropriate drugs recently published for the pediatric population [27]. Moreover, the study time was long enough to be able to draw conclusions. 

## 5. Conclusions

This study has shown different patterns in the drugs involved and the types of ADRs that occur according to the age of pediatric patients and highlights the need to assess ADRs in the pediatric population in hospital settings. Newborns, infants and toddlers were the group of patients with the highest number of serious and unlabeled ADRs, and also had more ADRs due to medication errors. In addition, off-label drugs involved in ADRs were more frequently reported in the childhood group—particularly in adolescents; therefore, more research on pediatric patients should be promoted and encouraged in order to have a higher level of efficacy and safety evidence in this population. These results should be interpreted with caution due to under-reporting and biases in the spontaneous reporting of ADRs.

In pediatric patients, spontaneous reporting is a valuable pharmacovigilance tool for assessing the safety of drugs, alongside other complementary methods. The results of our study suggest that data from hospital settings are important for generating signals and helping to identify opportunities for improving the safety of drugs used in the pediatric population. It is important that, to achieve this goal, hospital pharmacovigilance programs receive adequate resources to perform this function.

## Figures and Tables

**Table 1:** Classification of Adverse Drug Reactions according to Organ and System Classification and seriousness.

Organ and Systems Classification (SOC): N (%)	Seriousness: N (%)
Nervous system disorders: 14 (13.5) Seizure (4), dystonia (2), somnolence (2), encephalopathy (2), addiction (1), headache (1), hypotonia (1), optic neuritis (1)	12 (86)
Immune system disorders: 12 (11.7) Infusion reaction (7), anaphylactic shock (2), urticaria (2), erythema (1)	6 (50)
Skin and subcutaneous tissue disorders: 12 (11.7) Erythema or skin rash (9), angioedema (2), psoriasis (1)	0 (0)
Blood and lymphatic system disorders: 9 (8.7) Thrombocytopenia (3), neutropenia (3), haemorrhage (2), bone marrow aplasia (1)	6 (66.7)
Infections and infestations: 9 (8.7) Cellulitis (3), respiratory infection (2), herpes infection (1), otitis (1), hordeolum (1), subcutaneous infection (1)	5 (55.5)
Cardiac disorders: 8 (7.8) Arrhythmias (6), cardiac arrest (1), myocardiotoxicity (1)	5 (62.5)
Metabolic and nutritional disorders: 8 (7.8) Hypokalaemia (4), acidosis (1), alkalosis (1), hyperglycaemia (1), hypoglycaemia (1)	6 (75)
Gastrointestinal disorders: 7 (6.8) Pancreatitis (3), dysphagia (2), mucositis (1), perforated duodenal ulcus (1)	6 (85.7)
Psychiatric disorders: 4 (3.9) Agitation or irritability (2), anxiety (1), sleep disturbances (1)	1 (25)
Hepatobiliary disorders: 3 (2.9) Liver enzyme increases (3)	1 (33.3)
Renal and urinary disorders: 3 (2.9) Acute renal failure (2), nephrotic syndrome (1)	3 (100)
Traumatic injuries, intoxications and complications of therapeutic procedures: 3 (2.9) Intoxication (3)	3 (100)
General disorders and alterations at site of administration: 2 (1.9) Fever (1), fainting (1)	0 (0)
Endocrine disorders: 2 (1.9) Syndrome of inappropriate antidiuretic hormone (SIADH) (2)	2 (100)
Vascular disorders: 2 (1.9) Arterial hypertension (AHT) (1), cerebral venous thrombosis (1)	1 (50)
Congenital, familial and genetic disorders: 2 (1.9) Congenital cardiopulmonary malformation (1), cardiomyopathy (1)	2 (100)
Musculoskeletal disorders: 1 (1.0) Systemic lupus erythematosus (SLE) /Pericarditis (1)	1 (100)
Respiratory, thoracic and mediastinal disorders: 1 (1.0) Respiratory arrest (1)	1 (100)
Total: 103 (100)	61 (59.2)

**Table 2:** Involved drugs classified according to the Anatomical Therapeutic Chemical (ATC) classification system.

ATC Category	Therapeutic Area	N	%
L	Antineoplastic and immunomodulating agents: Methotrexate (7), adalimumab (5), azathioprine (5), doxorubicin (4), rituximab (4), vincristine (4), asparaginase (3), cyclophosphamide (3), tocilizumab (3), infliximab (2), antithymocyte immunoglobulin (1), cytarabine (1), PEG-asparaginase (1), siltuximab (1), tacrolimus (1)	45	32.4
N	Nervous system: Metamizole (3), oxcarbazepine (3), valproic acid (2), acetaminophen (paracetamol) (1), acetylsalicylic acid (1), methylphenidate (1), bupivacaine (1), caffeine (1), clonazepam (1), dextromethorphan (1), dimemorfan (1), droperidol (1), fentanyl (1), fluoxetine (1), haloperidol (1), lamotrigine (1), lidocaine (1), lorazepam (1), mepivacaine (1), olanzapine (1), petidine (1), quetiapine (1), remifentanil (1), sulpiride (1), ziprasidone (1)	30	21.6
J	Anti-infectives for systemic use: Amoxicillin-clavulanate (2), cefazolin (2), meningococcal ACWY vaccine (2), meningococcal B vaccine (2), ceftazidime (1), clindamycin (1), doxycycline (1), DTaP vaccine (1), ganciclovir (1), gentamicin (1), immunoglobulin (1), influenza vaccine (1), isoniazide (1), metronidazole (1), pneumococcal vaccine (1), valganciclovir (1), vancomicin (1), varicella vaccine (1)	22	15.9
R	Respiratory system: Salbutamol (5), salmeterol (2), budesonide (1), montelukast (1), vilanterol (1), fluticasone (1), ivy * (1)	12	8.6
M	Musculo-skeletal system: Ibuprofen (8), dexketoprofen (1)	9	6.5
H	Systemic hormonal preparations, excluding sex-hormones and insulins: Methylprednisolone (4), dexamethasone (2), corticotrophin (1)	7	5.0
A	Alimentary tract and metabolism: Ondansetron (3), insulin (1), pyridoxine (1), vitamin D (1)	6	4.3
C	Cardiovascular system: Bisoprolol (1), furosemide (1), epinephrine (1)	3	2.15
V	Various: Iomeprol (1), sugammadex (1), melissa officinalis * (1)	3	2.15
D	Dermatologicals: Isotretinoin (1)	1	0.7
S	Sensory organs: Cyclopentolate (1)	1	0.7
B	Blood and blood forming organs	0	0
G	Genito-urinary system and sex hormones	0	0
P	Antiparasitic products, insecticides and repellents	0	0
	Total	139	100

* Herbal products.

**Table 3:** Characteristics of adverse drug reactions (ADRs) due to medication errors.

Sex	Age	Drug	ADR	Type of Error	Seriousness
Female	21 days	Vitamin D	Intoxication	Overdose	Serious
Male	1 month	Acetaminophen	Hepatitis	Overdose	Serious
Female	1 month	Valproic acid	Somnolence	Overdose	Serious
Female	4 months	Dextromethorphan	Intoxication	Overdose	Serious
Male	8 years	Immunoglobulin	Cerebral venous thrombosis	Drug error	Serious
Female	10 years	Insulin	Hypoglycaemia	Overdose	Nonserious
Female	14 years	Valproic acid	Intoxication	Overdose	Serious
Female	17 years	Bisoprolol	AV blocking	Overdose	Nonserious

**Table 4:** Involved drugs with off-label use in adverse drug reactions (ADRs).

Sex	Age	Drug	Indication	ADR	Type of Off-Label	Severity
Female	4 months	Dextromethorphan	Cough	Intoxication *	Patient age	Serious
Male	8 months	Corticotrophin	Spasms	Hypokalaemia	Indication	Serious
Male	5 years	Pyridoxine	Convulsions	Inefficacy	Patient age	Serious
Male	5 years	Ondansetron	Nausea and vomiting	Cardiac arrest	Indication	Serious
Female	9 years	Ibuprofen	Pain	Infusional reaction	Patient age	Nonserious
Male	14 years	Ondansetron	Nausea and vomiting	Erythema	Indication	Nonserious
Male	16 years	Olanzapine	Anxiety	Bradycardia and ventricular trigeminy	Patient age Indication	Serious
Female	16 years	Sulpiride	Dizziness	Acute dystonia	Patient age Posology	Serious
Male	17 years	Siltuximab	Castelman disease	Thrombocytopenia	Patient age	Nonserious
Female	17 years	Bisoprolol	Palpitations/ ventricular extrasystoles	AV blocking	Patient age	Nonserious

*: Overdose of dextromethorphan due to a medication error.

**Table 5:** Comparison of adverse drug reactions (ADR) by patient age subgroup.

**ADR Characteristics**				
	**NIT** **(*n* = 27)**	**Child** **(*n* = 35)**	**Adolescent** **(*n* = 41)**	***p***
Male: *n* (%)	17 (63)	19 (54.3)	23 (56.1)	0.774
Type A ADR: *n* (%)	16 (59.3)	15 (42.9)	15 (36.6)	0.177
Serious ADR: *n* (%)	26 (96.3)	16 (45.7)	19 (46.3)	<0.001
Hospital-acquired ADR: *n* (%)	14 (51.9)	15 (42.9)	13 (31.7)	0.241
Number of involved drugs: median (IQR)	1 (1–2)	1 (1–1)	1 (1–1)	0.084
ADR by SOC category ^a^	Nervous system	Immune system disorders	Skin and subcutaneous tissue disorders	NA
**Involved drugs characteristics**				
	**NIT** **(*n* = 38)**	**Child** **(*n* = 47)**	**Adolescent** **(*n* = 54)**	***p***
Unlabeled reactions: *n* (%)	8 (21.1)	2 (4.3)	1 (1.9)	0.004
Drugs by ATC category ^b^	Nervous system (N)	Antineoplastic and immunomodulating agents (L)	Antineoplastic and immunomodulating agents (L)	NA

IQR: interquartile range; NA: not applicable; NIT: newborn, infant and toddler; *p*: *p* value, differences between three groups. ^a^ System Organ Classes (SOC) according to the MedDRA® dictionary. Groups were reported when proportion was >15%. ^b^ Categories of the Anatomical Therapeutic Chemical (ATC) classification System (level 1). Groups were reported when proportion was >25%.

## Data Availability

The data presented in this study are available on request from the corresponding author.
